# Cost-Effectiveness Analysis of 12-Versus 4-Weekly Administration of Bone-Targeted Agents in Patients with Bone Metastases from Breast and Castration-Resistant Prostate Cancer

**DOI:** 10.3390/curroncol28030171

**Published:** 2021-05-13

**Authors:** Megan M. Tu, Mark Clemons, Carol Stober, Ahwon Jeong, Lisa Vandermeer, Mihaela Mates, Phillip Blanchette, Anil Abraham Joy, Olexiy Aseyev, Gregory Pond, Dean Fergusson, Terry L. Ng, Kednapa Thavorn

**Affiliations:** 1 Cancer Therapeutics Program, Ottawa Hospital Research Institute, Ottawa, ON K1H 8L6, Canada; mtu@ohri.ca (M.M.T.); CSTOBER@ohri.ca (C.S.); ahwonjeong023@gmail.com (A.J.); lvandermeer@ohri.ca (L.V.); teng@toh.ca (T.L.N.); 2 Faculty of Medicine, University of Ottawa, Ottawa, ON K1H 8M5, Canada; 3 Division of Medical Oncology, Department of Medicine, The Ottawa Hospital, Ottawa, ON K1H 8L6, Canada; 4 Division of Medical Oncology, Cancer Centre of Southeastern Ontario and Queen’s University, Kingston, ON K7L 5P9, Canada; Mihaela.Mates@kingstonhsc.ca; 5 Division of Medical Oncology, Department of Oncology, London Regional Cancer Program, London Health Sciences Centre and University of Western Ontario, London, ON N6A 5W9, Canada; Phillip.Blanchette@lhsc.on.ca; 6 Division of Medical Oncology, Department of Oncology, University of Alberta, Edmonton, AB T6G 1Z2, Canada; Anil.Joy@albertahealthservices.ca; 7 Regional Cancer Care Northwest, Thunder Bay Regional Health Sciences Centre, Thunder Bay, ON P7B 6V4, Canada; aseyevo@tbh.net; 8 Department of Oncology, McMaster University, Hamilton, ON L8V 5C2, Canada; gpond@mcmaster.ca; 9 Clinical Epidemiology Program, Ottawa Hospital Research Institute, Ottawa, ON K1H 8L6, Canada; dafergusson@ohri.ca; 10 ICES, University of Ottawa, Ottawa, ON K1Y 4E9, Canada

**Keywords:** cost-effectiveness, bone metastasis, denosumab, pamidronate, zoledronate, breast cancer, prostate cancer

## Abstract

A cost–utility analysis was performed based on the Rethinking Clinical Trials (REaCT) bone-targeted agents (BTA) clinical trial that compared 12-weekly (once every 12 weeks) (*n* = 130) versus 4-weekly (once every 4 weeks) (*n* = 133) BTA dosing for metastatic breast and castration-resistant prostate (CRPC) cancer. Using a decision tree model, we calculated treatment and symptomatic skeletal event (SSE) costs as well as quality-adjusted life-years (QALYs) for each treatment option. Deterministic and probabilistic sensitivity analyses were performed to assess the robustness of the study findings. The total cost of BTA treatment in Canadian dollars (C$) and estimated QALYs was C$8965.03 and 0.605 QALY in the 4-weekly group versus C$5669.95 and 0.612 QALY in the 12-weekly group, respectively. De-escalation from 4-weekly to 12-weekly BTA reduces cost (C$3293.75) and improves QALYs by 0.008 unit, suggesting that 12-weekly BTA dominates 4-weekly BTA in breast and CRPC patients with bone metastases. Sensitivity analysis suggests high levels of uncertainty in the cost-effectiveness findings. De-escalation of bone-targeted agents is cost-effective from the Canadian public payer’s perspective.

## 1. Introduction

The optimal dosing interval for bone-targeted agents (BTA) in patients with cancer with bone metastases remains an important clinical question. As BTAs have no effects on either disease-free or overall survival, they are supportive care agents. For patients with bone metastases from breast cancer and castration-resistant prostate cancer (CRPC), their major benefit is reducing the frequency of and delaying the onset of symptomatic skeletal events (SSEs) while also improving patient health-related quality of life [1,2]. SSEs include new symptomatic pathological fractures, spinal cord compression, need for tumor-related orthopedic surgical intervention and radiotherapy to relieve bone pain, and sometimes hypercalcemia [3]. Rethinking Clinical Trials (REaCT) BTA (NCT02721433) was an open-label, multi-center, phase III trial that randomized 263 patients with metastatic breast cancer or CRPC to either 12-weekly (once every 12 weeks) or 4-weekly (once every 4 weeks) BTA with denosumab, pamidronate, or zoledronate for 1 year [4]. The study found that 12-weekly BTA treatment was non-inferior to 4-weekly BTA based on the primary outcome of change in patient reported health-related quality of life. This study is the largest prospective randomized, open label, clinical trial to date, involving patients with bone metastases from either breast or CRPC and comparing 12-Versus 4-weekly dosing of the three most commonly used BTAs [4]. Cost-effectiveness studies of BTA de-escalation have so far been based on the American payer system [5]. Shapiro et al. performed a cost-effectiveness analysis using a Markov model. Their analysis was based on the Cancer and Leukemia Group B/Alliance for Clinical Trials in Oncology (CALGB/Alliance) 70604 study, which explored the de-escalation of zoledronate to every 3 months. It therefore remains to be seen whether their findings will be applicable to patients who may receive other BTAs. To inform the decision to implement BTA de-escalation in Canada, we sought to determine the cost-effectiveness of 12-Versus 4-weekly BTA treatment from the perspective of Canada’s public healthcare system using the REaCT-BTA trial data.

## 2. Materials and Methods

We conducted a cost–utility analysis based on the published pragmatic, randomized, open-label, non-inferiority trial in patients with bone metastases from breast cancer or CRPC [4]. Patients from 5 Canadian centers (Ottawa, ON; London, ON; Kingston, ON; Thunder Bay, ON; and Edmonton, AB) were enrolled and randomized 1:1 to either the 12- or 4-weekly BTA treatment arms for 1 year. All 263 trial participants provided institutional review board-approved informed consent in accordance with institutional and national guidelines. The study is registered on clinicaltrials.gov (NCT02721433). The choice of which BTA was to be used—denosumab, pamidronate, or zoledronate—was made prior to randomization and was decided by the patient and their physician. In the clinical study, subgroup analyses were performed, with no significant differences in clinical outcomes by type of cancer or type of SSE identified. The costs and outcome measures used for this economic analysis were collected prospectively during the clinical trial (Table 1). The economic evaluation was performed from the perspective of Canada’s healthcare system.

### 2.1. Costs

All costs in the analysis are in Canadian dollars, based on 2019 values. A combination of data sources were used to estimate the costs used in this analysis (Table A1, Table A2, Table A3 and Table A4), including published Canadian sources [6] as well as the Ontario Case Costing Initiative (OCCI) [7], Cancer Care Ontario (CCO) [8], and Ontario Schedule of Benefits Physician Services [9]. We also consulted medical and radiation oncologists, orthopedic surgeons, and anesthesiologists at The Ottawa Hospital in Ottawa, Ontario, Canada to determine commonly used billing codes for physician services provided, and for surgical and anesthesia services. The average duration of the most common procedures for treating pathologic fractures (vertebral and non-vertebral) and spinal cord compression was estimated based on informal physician surveys and was rounded to the closest 15-min block (Table A2 and Table A3). The cost of each BTA used in this study—zoledronic acid 4 mg (DIN 024742805), pamidronate 90 mg (DIN 02249685), pamidronate 60 mg (DIN 0224551), pamidronate 30 mg (DIN 02244550), and denosumab 120 mg (DIN 02368153)—was based on the CCO Provincial Drug Reimbursement Program, published on 3 October 2019 (Table A1). Administration costs for pamidronate and zoledronate were assumed to include infusion and supply costs only. Cost of administration time and supplies were obtained from the Canadian Agency for Drugs and Technologies in Health (CADTH) which utilized data from a Canadian study comparing zoledronate and pamidronate [6,10] (Table A1). For denosumab, administration costs were obtained from the literature, where costs were estimated based on information supplied by the manufacturer for the breast cancer indication [6]. Costs for chemotherapy would be similar between patients receiving different BTAs and are therefore excluded from the analysis. The clinical trial measured 5 SSE outcomes: radiotherapy to relieve bone pain, new symptomatic pathological fracture (vertebral and non-vertebral), spinal cord compression, tumor-related orthopedic surgical intervention, and hypercalcemia. We calculated the weighted cost of the 5 SSEs for patients receiving standard of care 4-weekly and de-escalated 12-weekly BTA based on the proportion of patients experiencing each event reported in the trial data (Table A4). 

### 2.2. Outcomes

Quality-adjusted life years (QALYs) were used as the outcome data of interest. All patients in the trial were asked to complete EORTC QLQ-C30 quality of life cancer questionnaires. These patient responses were then used to derive health utility values. The EORTC QLQ-C30 quality of life cancer questionnaires at baseline, 12, 24, 36, and 48 weeks were converted to cancer-specific EORTC QLU-C10D utilities using the Canadian-based algorithm described by McTaggart-Cowan et al. [11]. These estimates were then used to calculate total QALY from baseline to 48 weeks for each patient enrolled in the trial.

### 2.3. Assumptions

The following assumptions were made for the analysis of the trial data. All 5 trial centers were assumed to have the same expertise and to have followed similar protocols in patient management. Only SSEs that occurred during the 1-year trial period were deemed relevant and included in the analysis. Since BTA does not affect disease progression, only SSEs are considered in the economic evaluation.

### 2.4. Analysis

**Figure 1:**
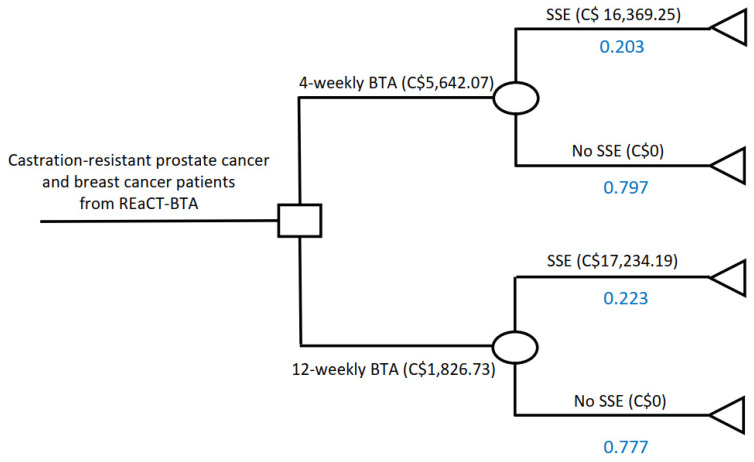
Decision tree comparing costs of 12-Versus 4-weekly BTA administration. REaCT = Rethinking Clinical Trials; BTA = bone-targeted agent; SSE = symptomatic skeletal event.

**Figure 2:**
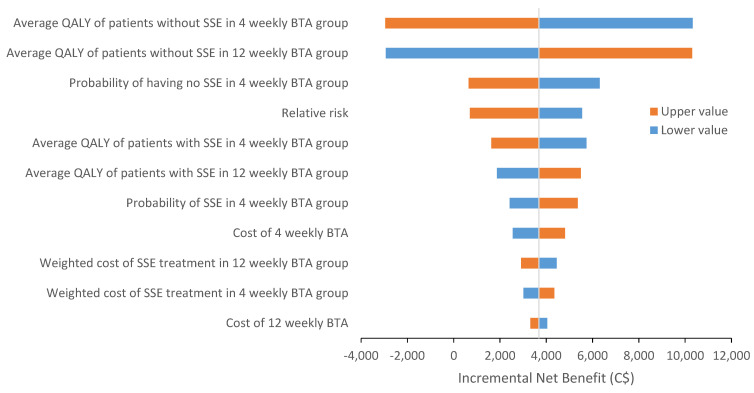
Tornado diagram of deterministic sensitivity analysis for costs associated with 12- and 4-weekly BTA treatment. QALY = quality-adjusted life year; BTA = bone-targeted agent; SSE = symptomatic skeletal event.

**Figure 3:**
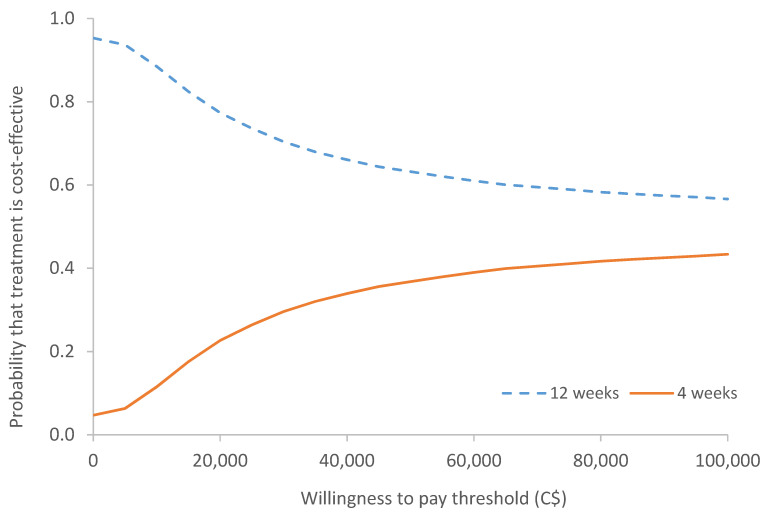
Cost-effectiveness acceptability curve (CEAC) for probabilistic model comparing 12-Versus 4-weekly BTA treatment showing the probability of 12-Versus 4-weekly BTA treatment being cost-effective at various willingness-to-pay thresholds.

A decision tree model was used to analyze the cost-effectiveness of 12-Versus 4-weekly BTA treatment (Figure 1). A 48-week time horizon was used to correspond with the length of the trial. As the time frame of this economic evaluation was only 1 year, discounting was deemed unnecessary since no adjustments for future costs and health outcomes were needed over this short timeframe. A deterministic sensitivity analysis was performed to assess the uncertainty associated with input parameters for the base case. The effect of changing a single parameter at a time and its impact on the overall ICER are summarized in a tornado diagram (Figure 2). In addition, a probabilistic sensitivity analysis of the base case was performed to explore the uncertainties in the cost and outcome data. A total of 5000 simulations were carried out using the Monte Carlo technique, the results of which are shown in a cost-effectiveness acceptability curve (Figure 3). The results of these analyses are presented as incremental cost effectiveness ratio (ICER) and incremental net benefit (INB) based on the widely utilized willingness-to-pay threshold of C$50,000 per QALY.

## 3. Results

The 4-weekly BTA group was associated with a total cost of C$8965.03 and 0.605 QALYs, while the 12-weekly BTA group was associated with C$5669.95 and 0.612 QALYs. In the base case analysis comparing only the differential costs of BTA treatment for 12-Versus 4-weekly (Table 2), 12-weekly dominated with respect to 4-weekly as this treatment option reduced cost (C$3293.75) and improved QALYs by 0.008 units. The incremental net benefit is C$3681.37 based on a willingness to pay of C$50,000 per QALY.

### Sensitivity Analysis

Results from deterministic sensitivity analyses suggested that the key factors which impacted on the results included changes in QALY of patients without SSEs, the probability of having no SSE, and the relative risk of developing SSE in 12-weekly BTA compared to 4-weekly BTA (Figure 2).

The cost-effectiveness acceptability curve derived from the probabilistic sensitivity analysis (Figure 3) shows that regardless of the willingness-to-pay threshold, there is a higher probability of 12-weekly BTA being more cost-effective than 4-weekly BTA. At the willingness-to-pay values of C$50,000 per QALY, the probability that 12-weekly BTA is cost-effective is 63.2%. However, as the willingness-to-pay values increase, the cost-effectiveness of 12-weekly BTA decreases since its advantage over 4-weekly BTA decreases.

## 4. Discussion

Our analysis showed that 12-weekly BTA was cost-effective compared to 4-weekly BTA since 12-weekly BTA was less expensive and marginally increased QALYs. The difference in costs between the 12- and 4-weekly schedules were mainly a result of differential drug costs: C$5642 for 4-weekly versus C$1827 for 12-weekly over the course of the 48-week clinical trial period. Since 12-weekly BTA used fewer resources and slightly increased QALY by 0.008, this treatment option was cost-effective when compared to a commonly used threshold of C$50,000 per QALY. The cost-effectiveness acceptability curve depicted the uncertainty around the cost effectiveness estimates based on the likelihood of 12-weekly BTA being cost effective at a given willingness-to-pay threshold compared to 4-weekly BTA. The observation of high uncertainty in the cost-effectiveness results could be due to small sample size (*n* = 130 for 12-weekly versus *n* = 133 for 4-weekly) and minimal differences in QALYs (0.008; 0.612 for 12-weekly versus 0.604 for 4-weekly).

### 4.1. Strengths and Limitations

Economic data were collected in the context of a randomized clinical trial, which enabled for more accurate estimation of cost and outcome while minimizing false clinical assumptions and selection bias. Since utilities were derived based on a conversion of patient responses from EORTC-QLQ-C30 to EORTC QLU-C10D, they were not directly measured from the study. Therefore, the derivation of the information is only as good as the algorithm used. We did however compare different derivation methods and found that EORTC QLU-C10D produced the best fit. This was determined by comparatively analyzing global health status using the conversion approaches proposed by McTaggart-Cowan et al. (C10D) [11], Crott and Briggs (EQ-5D) [12], and Rowen et al. [13], as outlined in the QLQ-C30 scoring manual [14]. These estimates were used to calculate total QALYs over 48 weeks for each patient in the study. The study only collected quality of life data during the first 48 weeks of the study. While this potentially impacts the level of confidence of our data, we believe that the cost-effectiveness of 12-weekly BTA will be sustained given the risk of SSE decreases over time [15]. Due to the limited sample size of 130 patients in the 12-weekly arm and 133 patients in the 4-weekly arm, we were unable to adequately assess differences between 12- and 4-weekly BTA across BTA type (i.e., denosumab vs. pamidronate vs. zoledronate) and cancer type (breast vs. prostate cancer). Due to the relatively small sample size, calculated weighted averages were used in the analysis and thus there could be variations amongst the different BTAs which we are unable to discern. Due to concerns about reducing denosumab frequency to every 3 months and its potential risk of rebound in bone health deterioration [16], the results of the more definitive REDUSE trial are eagerly awaited [17]. Despite this, surveys of health care providers confirm that the 12-weekly treatment regimen is becoming increasingly common [18]. Other limitations are that the included patients started BTAs at any time and not just at baseline; this is important as longer durations of BTA use appear to be associated with reduced SSEs and increased risk of serious toxicities [15].

### 4.2. Interpretation Considering Other Studies

This is the first study to prospectively compare cost–utility of 12-Versus 4-weekly BTA treatment in patients with metastatic cancer from a Canadian payer perspective. Previously published economic evaluations involving the use of BTAs in patients with metastatic cancer have focused on the cost of denosumab versus bisphosphonates, with the majority of data based on a US payer perspective [19,20,21,22,23].

Shapiro et al. performed cost-effectiveness analysis on the results from the CALGB/Alliance 70604 study, a randomized clinical trial which showed that zoledronate every 3 months was non-inferior to zoledronate every month in reducing the risks of skeletal-related events [5]. Using a Markov model, their cost-effectiveness analysis (performed from the US payer perspective) concluded that zoledronate every 3 months dominated monthly zoledronate and denosumab. It should be noted that the CALGB/Alliance 70604 clinical trial did not look at the de-escalation of denosumab, so were unable to compare the cost-effectiveness of zoledronate versus denosumab every 3 months. However, these results were in line with our findings from the REaCT-BTA clinical trial in which de-escalated treatment with 12-weekly BTA dominated 4-weekly BTA in terms of cost effectiveness.

## 5. Conclusions

Based upon our analysis, de-escalation of BTA treatment from 4-weekly to 12-weekly is cost-effective. For patients with CRPC and breast cancer with bone metastases, 12-weekly BTA treatment is a reasonable clinical choice as it has been found to be clinically non-inferior [4] as well as more cost-effective in the Canadian healthcare setting. Future work could be done to expand the analyzable data with a larger cohort which would allow for an individual analysis of the different BTA treatments, especially bisphosphonates versus denosumab, as well as comparisons between different cancer types.

## Figures and Tables

**Table 1:** Input parameters used in the model.

	Baseline	Lower limit	Upper limit	Reference
**Probabilities**				
Probability of SSE with 4-weekly BTA	0.203	0.1338	0.2954	[4]
Probability of no SSE with 4-weekly BTA	0.797	0.6525	0.9639	[4]
Probability of SSE with 12-weekly BTA	0.223			[4]
Probability of no SSE with 12-weekly BTA	0.777			[4]
**De-escalation effect (relative risk)**				
Reduction of BTA treatment from 4-weekly to 12-weekly	1.099	0.6902	1.7496	[4]
**Costs per year**				
Weighted cost of 4-weekly BTA	C$5642.07	C$4513.66	C$6770.48	Appendix A
Weighted cost of 12-weekly BTA	C$1826.73	C$1461.38	C$2192.08	Appendix A
Weighted cost of SSE in 4-weekly BTA group	C$16,369.25	C$13,095.40	C$19,643.10	Appendix B
Weighted cost of SSE in 12-weekly BTA group	C$17,234.19	C$13,787.35	C$20,681.03	Appendix B

BTA = bone-targeted agent; SSE = symptomatic skeletal event.

**Table 2:** Cost-effectiveness results.

	Costs	QALY
4-weekly BTA	C$8965.03	0.605
12-weekly BTA	C$5671.28	0.612
Incremental	−C$3293.75	0.008
ICER (∆ cost/∆ QALY)		12-weekly dominates 4-weekly
Incremental net benefit (INB) *		C$3681.37
* The INB is based upon an assumption that the willingness to pay for one QALY is C$50,000		
If INB > 0 = intervention is cost effective		
If INB < 0 = not cost effective		

QALY = quality-adjusted life year; BTA = bone-targeted agent; ICER = incremental cost-effectiveness ratio. The asterisk denotes a footnote to clarify that the incremental net benefit (INB) is based on upon an assumption of willingness to pay of C$50,000 for one QALY.

## Appendix A

**Table A1:** Drug costs.

	4-Weekly			12-Weekly		
	Cost of Drug (C$) [8]	Cost of Administration (C$) [6]	Total Cost (C$)	Cost of Drug (C$) [8]	Cost of Administration (C$) [6]	Total Cost (C$)
Denosumab (Xgeva)	7387.34	372.00	7759.34	2462.45	124.00	2586.45
Pamidronate	130.68	227.64	358.32	43.56	975.88	1019.44
Zoledronate	96.72	2370.00	266.72	32.24	790.00	822.24

## Appendix B

**Table A2:** SSE costs.

Variable	Value (C$)	Source
**Radiotherapy to relieve bone pain**		
Average cost of external beam radiotherapy to axial skeleton (1AW27JA,1EA27JA,1SQ27JA)	18,223	[7]
Radiation oncologist—consultation (A345)	152.40	[9]
Radiation oncologist—radiation treatment planning—level 3 (X312)	680.45	[9]
Total cost of radiotherapy to relieve bone pain	**19,055.85**	
**Pathological fracture**		
Average cost of pathological fracture, pelvic region and thigh (M8445)	4890	[7]
Orthopedic surgeon—consultation (A065)	83.10	[9]
Orthopedic surgeon—fractures—open reduction (F096)—“femoral nail fixation” = $493.80	492.38	[9]
Orthopedic surgeon—fractures—open reduction—primary prosthesis, femur only (F101) “partial hip replacement for neck fracture” = $490.95		[9]
Radiation oncologist—radiation treatment planning—level 3 (X312)	680.45	[9]
Anesthesiologist (see Appendix C for cost breakdown)	405.27	[9]
Total cost to treat pathological fracture	**6145.93**	
**Spinal cord compression**		
Average cost of cord compression (G952)	6496	[7]
Radiation oncologist—consultation (A345)	152.40	[9]
Radiation oncologist—radiation treatment planning—level 3 (X312)	680.45	[9]
Total cost to treat spinal cord compression	**7328.85**	
**Tumor-related orthopedic surgical intervention**		
Average cost of prophylactic surgery (Z408, Z409)—day surgery	2375	[7]
Orthopedic surgeon—consultation (A065)	83.1	[9]
Orthopedic tumor surgery—biopsy of suspected sarcoma, or resection of a complex bone or complex soft tissue	600	[9]
tumor(s), $100 per 15 min (R226A), assume 90min—“general oncology”		
Orthopedic surgeon—bone—major tumor resection (R330)—“tumor excision”	629.65	[9]
Average cost of the two surgeries	614.83	[9]
Anesthesiologist (see Appendix C for cost breakdown)	405.27	
Total cost for tumour-related orthopedic surgical intervention	**3478.195**	
**Hypercalcemia**		
Average cost of disorder of calcium metabolism (E835)	7448	[7]
Medical oncologist—consultation (A445)	157	[9]
Total cost to treat hypercalcemia	**7605**	

## Appendix C

**Table A3:** Anesthesiology costs [9].

Description	Base price [9]	Units	Total	Average
**Pathological fracture**				C$405.27
**F096 (75–90 min (use 90 min) + 30 min pre/post-op)**	C$15.01			
To start case		8	C$120.08	
First 60 min (1 unit per 15 min)		4	C$60.04	
60–90 min (2 units per 15 min)		4	C$60.04	
>90 min (4 units per 15 min)		8	C$120.08	
ASAII (E022C × 2 units)		2	C$30.02	
			**C$390.26**	
**F101 (75–90 min (use 90 min) + 30 min pre/post op)**	C$15.01			
To start case		10	C$150.10	
First 60 min (1 unit per 15 min)		4	C$60.04	
60–90 min (2 units per 15 min)		4	C$60.04	
>90 min (4 units per 15 min)		8	C$120.08	
ASAII (E022C × 2 units)		2	C$30.02	
			**C$420.28**	
**Tumor-related orthopedic surgical intervention**				C$405.27
**R226A (45 min–24 h (use 90 min) + 30 min pre/post op)**	C$15.01			
To start case		15	C$225.15	
First 60 min (1 unit per 15 min)		4	C$60.04	
60–90 min (2 units per 15 min)		4	C$60.04	
> 90 min (4 units per 15 min)		8	C$120.08	
			**C$465.31**	
**R330**	C$15.01			
To start case		7	C$105.07	
First 60 min (1 unit per 15 min)		4	C$60.04	
60–90 min (2 units per 15 min)		4	C$60.04	
>90 min (4 units per 15 min)		8	C$120.08	
			**$345.23**	

## Appendix D

**Table A4:** SSE incidence and weighted costs.

SSE	Number of Events [Proportion of Total Events]		Weighted Cost (C$) of Each SSE	
	4-Weekly	12-Weekly	4-Weekly	12-Weekly
Radiotherapy to bone	35 [0.78]	38 [0.86]	14,821.22	16,457.33
Hypercalcemia	4 [0.089]	1 [0.023]	676.00	172.84
Pathological fracture	4 (0.089)	2 [0.045]	546.30	279.36
Spinal cord compression	2 [0.044]	1 [0.023]	325.73	166.56
Surgery to bone	0 [0]	2 [0.045]	0.00	158.10
Total	45	44	16,369.25	17,234.19

## References

[1] Clemons M., Gelmon K., Pritchard K., Paterson A. (2012). Bone-targeted agents and skeletal-related events in breast cancer patients with bone metastases: The state of the art. Curr. Oncol..

[2] Van Poznak C., Somerfield M.R., Barlow W.E., Biermann J.S., Bosserman L.D., Clemons M.J., Dhesy-Thind S.K., Dillmon M.S., Eisen A., Frank E.S. (2017). Role of Bone-Modifying Agents in Metastatic Breast Cancer: An American Society of Clinical Oncology–Cancer Care Ontario Focused Guideline Update. J. Clin. Oncol..

[3] Von Moos R., Costa L., Scagliotti G., Sleeboom H., Goldwasser F., Hirsh V., Spencer A., Radcliffe H.-S., Niepel D., Henry D. (2016). Symptomatic skeletal events (SSEs) versus skeletal-related events (SREs) in patients with advanced cancer and bone metastases treated with denosumab or zoledronic acid. Ann. Oncol..

[4] Clemons M., Ong M., Stober C., Ernst S., Booth C., Canil C., Mates M., Robinson A., Blanchette P., Joy A.A. (2021). A randomised trial of 4- versus 12-weekly administration of bone-targeted agents in patients with bone metastases from breast or castration-resistant prostate cancer. Eur. J. Cancer.

[5] Shapiro C.L., Moriarty J.P., Dusetzina S., Himelstein A.L., Foster J.C., Grubbs S.S., Novotny P.J., Borah B.J. (2017). Cost-Effectiveness Analysis of Monthly Zoledronic Acid, Zoledronic Acid Every 3 Months, and Monthly Denosumab in Women with Breast Cancer and Skeletal Metastases: CALGB 70604 (Alliance). J. Clin. Oncol..

[6] (2016). CADTH Common Drug Reviews. Denosumab (Xgeva).

[7] (2019). Ontario Case Costing Initiative (OCCI). https://data.ontario.ca/dataset/ontario-case-costing-initiative-occi.

[8] (2019). Drugs Reimbursed by the Provincial Drug Reimbursement Programs (PDRP).

[9] Schedule of Benefits. Physician Services Under the Health Insurance Act. 1 March 2016. http://www.health.gov.on.ca/en/pro/programs/ohip/sob/physserv/sob_master20160401.pdf.

[10] Dranitsaris G., Castel L.D., Baladi J.F., A Schulman K. (2001). Zoledronic acid versus pamidronate as palliative therapy in cancer patients: A Canadian time and motion analysis. J. Oncol. Pharm. Pract..

[11] McTaggart-Cowan H., King M.T., Norman R., Costa D.S.J., Pickard A.S., Regier D.A., Viney R., Peacock S.J. (2019). The EORTC QLU-C10D: The Canadian Valuation Study and Algorithm to Derive Cancer-Specific Utilities from the EORTC QLQ-C30. MDM Policy Pract..

[12] Crott R., Briggs A. (2010). Mapping the QLQ-C30 quality of life cancer questionnaire to EQ-5D patient preferences. Eur. J. Health Econ..

[13] Rowen D., Brazier J., Young T., Gaugris S., Craig B.M., King M.T., Velikova G. (2011). Deriving a preference-based measure for cancer using the EORTC QLQ-C30. Value Health.

[14] Aaronson N.K., Ahmedzai S., Bergman B., Bullinger M., Cull A., Duez N.J., Filiberti A., Flechtner H., Fleishman S.B., De Haes J.C. (1993). The european organization for research and treatment of cancer qlq-c30: A quality-of-life instrument for use in international clinical trials in oncology. J. Natl. Cancer Inst..

[15] Ng T.L., Tu M.M., Ibrahim M.F.K., Basulaiman B., McGee S.F., Srikanthan A., Fernandes R., VanderMeer L., Stober C., Sienkiewicz M. (2021). Long-term impact of bone-modifying agents for the treatment of bone metastases: A systematic review. Support. Care Cancer.

[16] Lamy O., Gonzalez-Rodriguez E., Stoll D., Hans D., Aubry-Rozier B. (2017). Severe Rebound-Associated Vertebral Fractures After Denosumab Discontinuation: 9 Clinical Cases Report. J. Clin. Endocrinol. Metab..

[17] Gillessen S., von Moos R.A.F., Hayoz S., Hawle H., Cathomas R., Rothermundt C.A., Anchisi S., Mueller A., Wehrhahn T., Burmeister H. (2019). Incidence of hypocalcemia in patients with castration-resistant prostate cancer treated with denosumab: Data from a non-inferiority phase III trial assessing prevention of symptomatic skeletal events (SSE) with denosumab administered every four weeks (q4w) versus every 12 weeks (q12w)—SAKK 96/12 (REDUSE). J. Clin. Oncol..

[18] AlZahrani M., Clemons M., Vandermeer L., Sienkiewicz M., Awan A.A., Hutton B., Pond G.R., Ng T.L. (2021). Real-world practice patterns and attitudes towards de-escalation of bone-modifying agents in patients with bone metastases from breast and prostate cancer: A physician survey. J. Bone Oncol..

[19] Xie J., Namjoshi M., Wu E.Q., Parikh K., Diener M., Yu A.P., Guo A., Culver K.W. (2011). Economic Evaluation of Denosumab Compared with Zoledronic Acid in Hormone-Refractory Prostate Cancer Patients with Bone Metastases. J. Manag. Care Pharm..

[20] Xie J., Diener M., Sorg R., Wu E.Q., Namjoshi M. (2012). Cost-effectiveness of denosumab compared with zoledronic acid in patients with breast cancer and bone metastases. Clin. Breast Cancer.

[21] Snedecor S.J., Carter J.A., Kaura S., Botteman M.F. (2012). Cost-effectiveness of denosumab versus zoledronic acid in the management of skeletal metastases secondary to breast cancer. Clin. Ther..

[22] Stopeck A., Rader M., Henry D., Danese M., Halperin M., Cong Z., Qian Y., Dansey R., Chung K. (2012). Cost-effectiveness of denosumab vs zoledronic acid for prevention of skeletal-related events in patients with solid tumors and bone metastases in the United States. J. Med. Econ..

[23] Snedecor S.J., Carter J.A., Kaura S., Botteman M.F. (2013). Denosumab versus zoledronic acid for treatment of bone metastases in men with castration-resistant prostate cancer: A cost-effectiveness analysis. J. Med. Econ..

